# Editorial: Microbial communities of traditional fermented beverages: physiology, metabolism and interactions in fermentative processes

**DOI:** 10.3389/fmicb.2024.1480982

**Published:** 2024-09-03

**Authors:** Jesús Bernardo Páez-Lerma, Anne C. Gschaedler-Mathis, Christian Ariel Lopes

**Affiliations:** ^1^TECNM/Instituto Tecnológico de Durango Departamento de Ingenierías Química y Bioquímica, Durango, Mexico; ^2^Unidad de Biotecnología Industrial, Centro de Investigación y Asistencia en Tecnología y Diseño del Estado de Jalisco A.C., Zapopan, Mexico; ^3^Instituto de Investigación y Desarrollo en Ingeniería de Procesos y Biotecnología (PROBIEN), CONICET-Universidad Nacional del Comahue, Neuquén, Argentina

**Keywords:** traditional process, native yeast, population dynamics, fermentation, functional compounds

The interest in preserving traditional fermentation processes that give products a regional character and differentiate them by their unique characteristics is on the rise. It is crucial that these traditional processes, together with the microorganisms involved in their production, be adequately described and studied. This will not only position the products but also open the possibility of using these organisms in favorable environments to obtain products with less variability.

Therefore, it is essential to perform studies that explain the physiology and genetics of microorganisms, their capacity to adapt to diverse conditions, and the dynamics of fermentation in various traditional processes.

Among the yeasts, those belonging to the *Saccharomyces cerevisiae* species have been historically described in both commercial and traditional fermentation processes, due to their excellent fermentative ability. However, numerous reports have already described the presence and importance of other yeast species defined as non-*Saccharomyces*, as well as their interaction with different bacterial and fungal species in fermented beverages, especially those with little or no human intervention through the use of commercial starter cultures. Some of these are ethnic products with global cultural and historical significance. There is a growing interest in preserving traditional processes and their natural microbial communities.

Therefore, the main aim of this Research Topic was to present different studies about the characterization of microorganisms in traditional fermented beverages, including their diversity, interactions, and physiological and genetic features. Eight articles have been published, adding to our knowledge of these processes. A review by Roselli et al. described the importance of no- and low-alcohol (NoLo), alternatives to traditional alcoholic products, and microorganisms throughout the brewing process, including both their positive contributions and their negative (spoilage) effects, as well as the spoilage risks associated with these products, and how the microbiota can impact each product stream in terms of microbiological stability and final beer quality. Another review by Su et al. focused on a Kombucha tea-based beverage produced by fermentation with a symbiotic culture of bacteria, yeast, tea, and sugar water, describing the biological transformation pathways of kombucha metabolites and alternative substrates in detail. Also, this Topic Research includes five studies from several parts of the world and different applications of microbiota in the process, considering that bacteria isolated from Pulque have a functional effect, which is reported by Ruiz-Ramirez et al. who described Pulque as a traditional Mexican non-distilled alcoholic beverage, which is attributed several beneficial functions, mainly associated with gastrointestinal health: these could be explained by the presence of probiotic bacteria in its microbiota. Moghimani et al. contributed to a study on the use of bacteria and yeast in the fermentation of the Kefir beverage, which contains beneficial microorganisms that have health-promoting properties; therefore, Kefir has excellent potential to be a probiotic. Their study evaluated the probiotic potential and technological and safety characteristics of *Enterococcus faecalis, Lactococcus lactis*, and *Pichia fermentans* isolated from traditional kefir beverages. Cheng et al. described the interactions during the ultra-long fermentation of compound-flavor Baijiu and the variation of physicochemical parameters, microbial communities, metabolism, composition, and the proportion of volatile components in fermented grains. The main microorganisms found in baijiu and their impact on the organoleptic substances they create were described by Zhang et al.. Selvaraj and Gurumurthy demonstrated the predominant microbial species associated with Kombucha fermentation, including both bacteria and yeasts. These microorganisms include *Komagataeibacter rhaeticus, Gluconobacter oxydans, Brettanomyces bruxellensis*, and *Zygosaccharomyces bailli*, which give Kombucha its distinctive flavor profile. Finally Moreira-Ramos et al., proposed the use of different local *Saccharomyces cerevisiae* yeast strains to produce beers that have distinct organoleptic properties.

In conclusion, traditional fermentation processes are not just about the science of fermentation; they are associated with the traditions and culture of the communities in which they are developed. These processes produce accepted products with their own unique organoleptic characteristics and, in some cases, provide health benefits to those who consume them. This Research Topic has successfully combined these processes in a space where they are made known and has provided an intellectual contribution to the scientific community.

